# Hospitals and the Poets.—III. Keats' Seven Years of Medicine

**Published:** 1916-02-05

**Authors:** 


### HOSPITALS AND THE POETS.*

### III. Keats' Seven Years of Medicine.

The most famous poet who ever embarked upon a medical career, to leave it like his predestined comrades of the craft for literature, is John Keats. Yet it would be hard to find anywhere in his verse an indication that he had studied medicine or walked the wards of the London hospitals. We must content ourselves, therefore, with a brief sketch of those short years in his too short life during which he made a study of medicine.

Born in 1795, three years after Shelley, John Keats was the son of a London ostler in a successful way of business, who married the daughter of the man who owned the mews or stables where he worked. It is recorded, and may interest those who seek to explain the appearance of genius by heredity or other physical cause, that the poet was a seven months' child. At school Keats was said to be full of spirits and to be possessed of a fighting if hardly pugnacious, temperament. In 1809, when he was fourteen years old, Keats' mother fell into a decline, and her death in the following year was probably a contributory cause to Keats' apprenticeship to medicine, since, indirectly, it led to him and his brother being placed in the hands of two guardians, with the result that Keats was removed from school and articled, with a five years' apprenticeship, to Surgeon Hammond, at Edmonton. The impression which he made upon his fellow-students at Hammond's has been preserved for us. They described him as "an idle, loafing fellow always writing poetry." This want of sympathy between Keats and his surroundings evidently persisted and increased, since after four years—in 1814—a quarrel occurred between him and Mr. Hammond, which ended, as it was bound to end, in his indentures being dissolved by mutual consent. In the same year died Keats' grandmother, Mrs. Jennings, to whose solicitude the deed of trust appointing the two guardians of the orphan boys was due, and Keats became more than ever dependent upon the active guardian, Mr. Abbey.

### Keats in London.

Still with a medical career in prospect, Keats now moved to London and entered as a medical student at the combined school associated with St. Thomas' and Guy's, which for teaching purposes were then united. He lodged first in the Borough, and then in St. Thomas' Street, with two fellow-students with whom he became intimate. He now attended lectures and seems to have shown ability, but was an erratic worker, since his thoughts were fixed on Poetry, who had already marked him for her own in the very real sense of endowing him, in the following year, 1815, with power to write the earliest of his since famous poems—namely, the sonnet "On first looking into Chapman's Homer." That he was not content with his student life may be judged from the "Epistle to George Felton Mathew," afterwards an official of the Poor-Law Board, who has supplied reminiscences of Keats' life at this period. In that epistle—which, by the way, falls far short in poetic power of Shelley's "Letter to Maria Gisborne," with which it excites comparison—Keats says that he wishes he could follow his friend into poetry.

> But 'tis impossible; far different cares
> Beckon me sternly from soft "Lydian airs."
> And hold my faculties so long in thrall.

Keats was fond of writing such "epistles," and as another of them describes very summarily his detestation of soldiers, it is worth while to insert the passage here:

> On one side is a field of drooping oats,
> Through which the poppies show their scarlet coats,
> So pert and useless, that they bring to mind
> The scarlet coats that pester human kind.

### Poet and Practitioner.

In 1816 Keats had the misfortune of getting to know Leigh Hunt, with the result that the stimulus that he gained from Hunt's friendship had to be paid for heavily a little later, when the odium in which Hunt was held by the principal critics descended on his *protégé*, some of whose poems, including his first printed lines, "O Solitude, if I must with thee dwell," had appeared in Hunt's paper, the *Examiner*. On March 3 in this year Keats became a dresser at Guy's under Mr. Lucas, and on July 25 he qualified by passing his examination as a Licentiate of the Society of Apothecaries. Thus when *Endymion* appeared in 1818, it would have been possible for its title-page to have been inscribed "A poetic romance by John Keats, M.L.S.S.A., L.S.A.," after the manner of those successful professional men who seek to recommend their excursions into general literature by drawing attention to the degrees or decorations which have been awarded them when otherwise engaged. But *Endymion* was not published till two years after his qualification. In this year also Keats left the rooms near the hospital and spent some time at Leigh Hunt's cottage in the Vale of Health, Hampstead, where a bed in the library was kept for him. The fruit of this friendship, as already noted of doubtful advantage to Keats, was an article on his work by Leigh Hunt in the *Examiner*. As the year 1816 drew to a close, Keats resolved to give up the practice of surgery—a phrase we are entitled to use if we follow the biographer's account of "Some operations successfully performed" by him!

Having some small private means of his own, he was able to carry out this intention, and in the following March 1817 his *Poems* appeared, dedicated, as could only be expected, to Leigh Hunt. The publisher was Ollier. There was no sale; but Keats, on general grounds, had no great cause for depression, since Taylor and Hessey, who were

* The first article in this series appeared on page 329 of The Hospital for January 8, and the second on page 369 for January 22.

publishers of the *London Magazine*, not only accepted *Endymion*, on which he was now engaged, but even allowed Keats to draw advanced fees or royalties from them. He spent the summer at a house in Well Walk, Hampstead, and, apparently from mixed motives (including, it is said, a sense of shame at their difference of birth), declined Shelley's invitation to stay with him at Marlow, in spite of the fact that a tablet on the house, put up by the late Sir William Clayton, Bart., records the visit! During the autumn he was at Oxford, corresponding with Fanny Brawne, and in November he went to Burford Bridge, almost at the foot of Box Hill, already famous for Nelson's stay there on his last night in England, and since Keats' time celebrated as the residence of another great poet, Meredith. Here, as all of us have heard, *Endymion* was completed.

In 1818 Keats was nursing his brother Tom at Teignmouth, from which the two prefaces to *Endymion* are dated. The tour in Scotland followed. Keats' health began to give way, and at the end of the year his brother died. Then came 1819, the year of the marvellous odes, which were published in his third and final volume in 1820. At this time, too, Keats became involved in money troubles, the extent of which can be judged from the fact that he seriously contemplated taking up surgery once more at Edinburgh, or, as an alternative plan, of seeking the post of surgeon on board ship. His friend Brown influenced him successfully to abandon the project, and, like a true friend, lent him money. In 1820 his health definitely gave way; unmistakable symptoms of consumption supervened, and, as has already been hinted, the brutal criticism which his poems received, culminating with the gibe that he would be better employed in going back to his pills and his plasters, certainly accelerated a crisis of the nerves. He was advised to seek a warmer climate, and after a fearful voyage, arrived in Italy to die in Rome on February 13, 1821. We cannot but think of him and his own fate when we read in the ode *To a Nightingale* of his desire to forget the weariness and fever of this life "where youth grows pale and spectre-thin and dies"; which sums up with terse accuracy the ravages of the disease which killed him. That he had, like a true poet, that keenness of observation which after all is the quality, directed to clinical signs, that makes the good diagnostician, can be sufficiently shown by giving two lines of his descriptive of sleeplessness:

> We put our eyes into a pillowy cleft,
> And see the spangly gloom froth up and boil.

The physical symptoms of passion are once or twice sharply insisted on in his poems, and observation and physical sensation were so much blended in him that he could truly say, in a vivid foretaste of death, "I feel the flowers growing over me."

